# Ischemic preconditioning with active reperfusion does not improve high-intensity intermittent exercise in youth team sport players

**DOI:** 10.1007/s00421-025-06103-7

**Published:** 2026-01-12

**Authors:** Gustavo R. Mota, Izabela A. Santos, Anderson Luiz Rodrigues, Bernardo N. Ide, Tom Citherlet, Jeffer E. Sasaki, Moacir Marocolo

**Affiliations:** 1 Exercise Science, Health and Human Performance Research Group, Department of Sport Sciences, Institute of Health Sciences, Federal University of Triângulo Mineiro, Uberaba, Brazil; 2 Exercise Physiology in Health and Human Performance Research Group, Department of Physical Education, University of Uberaba (UNIUBE), Uberaba, Brazil; 3 Institute of Sport Sciences, University of Lausanne, Lausanne, Switzerland; 4 Integrated Laboratory of Physiology and Performance (LABIFID), Department of Biophysics and Physiology, Federal University of Juiz de Fora, Juiz de Fora, Brazil; 5 Department of Kinesiology, University of Wisconsin-Madison, Madison, WI, USA

**Keywords:** Blood flow, Endurance, Ergogenic, Soccer, Basketball, Ischemia

## Abstract

**Purpose:**

We investigated whether an active protocol of ischemic preconditioning (IPC-A) would improve high-intensity intermittent exercise performance in youth team sport players.

**Methods:**

Fifteen male amateur team sport players (15.5 ± 0.5 yrs) attended four different preconditioning sessions before the YoYo Intermittent Recovery Test level 1 (YYIR1) interspersed by seven days in a counterbalanced randomized cross-over design. IPC protocol consisted of three cycles of 5 min occlusion (220 mmHg) and 5 min reperfusion (0 mmHg) in both thighs. SHAM was similar to the IPC protocol, but ‘occlusion’ pressure was set up at 20 mmHg. Active protocols (IPC-A/ SHAM-A) were similar to the IPC/SHAM, but participants exercised (intermittent run) during the ‘reperfusion’ phases instead of resting. Six minutes after the protocol, the participants performed the YYIR1.

**Results:**

The distance covered in the YYIR1 did not differ (*p* = 0.46) among the protocols: IPC (917 ± 204 m) vs. IPC-A (931 ± 211 m) vs. SHAM (968 ± 201 m) vs. SHAM-A (933 ± 204 m). Blood lactate concentration, and mean heart rate did not differ either (*p* > 0.05) among the protocols.

**Conclusions:**

Active ischemic preconditioning involving exercise during reperfusion phase does not improve high-intensity intermittent exercise performance nor alter physiological or perceptual responses in youth team sport players.

## Introduction

Among elite athletes, small performance improvements in neuromuscular function are of major relevance, as even minor enhancements can exert a decisive impact in real-world contexts. In this context, ischemic preconditioning (IPC)—an intervention involving cycles of occlusion and reperfusion of blood flow using tourniquets at the proximal parts of limbs—has been extensively tested in various settings due to its feasibility and relative simplicity (Incognito et al. 2016; Marocolo et al. 2019).

The precise mechanisms underlying the potential enhancement of exercise performance through IPC remain unclear. However, factors such as reactive hyperemia and increased muscle oxygen extraction may provide justification for the observed benefits on muscle function (Andreas et al. 2011; Kido et al. 2015; Marocolo et al. 2023). A study demonstrated that during reperfusion, blood flow in IPC protocol was 1.1 and 8 times greater in mean and peak values, respectively, compared to the SHAM protocol (Mota et al. 2020). Furthermore, IPC has been shown to attenuate ATP depletion, increased phosphocreatine resynthesis, and oxygen uptake during the reperfusion phase (Andreas et al. 2011). Additionally, Kido et al. showed that IPC accelerated muscle deoxygenation dynamics during moderate-intensity exercise and improved endurance during severe-intensity exercise, attributing these effects to IPC-induced mitochondrial activity in skeletal muscle (i.e., higher activity to meet increased energy demands), as evidenced by accelerated oxygen extraction (Kido et al. 2015).

Despite the potential advantages, the effects of IPC on exercise performance improvements have yielded conflicting results. While some studies reported positive outcomes (Incognito et al. 2016), others demonstrated neutral effects, highlighting the absence of an established ideal protocol (Marocolo et al. 2019). Consequently, there is a need to explore new combinations or protocols and assess their efficacy in enhancing acute exercise performance. Previous studies investigating IPC have mainly examined cyclic efforts such as running, swimming, and cycling (Marocolo et al. 2016; Incognito et al. 2016; da Mota et al. 2019). However, these activities differ from high-intensity intermittent efforts, which are more predominant in team sports (Bangsbo et al. 2008). While research on the effects of IPC in intermittent efforts remains limited, IPC has shown promising results in high-intensity cyclic efforts—such as running or cycling—that involve sustained movement and rely on both aerobic and anaerobic metabolism (Cruz et al. 2015; Incognito et al. 2016). Nonetheless, the applicability of these findings to high-intensity intermittent exercise remains uncertain, particularly due to the unique demands of intermittent efforts, which typically involve repeated accelerations, decelerations, and changes of direction. These mechanical and neuromuscular characteristics may influence the physiological response to IPC and deserve further investigation. Although IPC has been widely studied in adults, its effects in adolescents remain unclear. Due to maturational differences in muscle metabolism and exercise responses, adolescents may respond differently to IPC in terms of physiological and performance outcomes (Armstrong et al. 2015).

Warm-up exercises, routinely performed before training or competition, are well established to enhance subsequent performance by increasing muscle temperature, improving cardiovascular and metabolic efficiency, facilitating neural activation, and promoting psychological readiness (McGowan et al. 2015). These integrated physiological adjustments accelerate oxygen uptake kinetics and optimize neuromuscular function, thereby priming the body for exercise. Interestingly, several of these responses parallel those elicited during the reperfusion phase of IPC, when transient elevations in blood flow and muscle oxygen delivery occur (Kido et al. 2015; McGowan et al. 2015; Mota et al. 2020). However, conventional IPC protocols are typically passive—performed entirely at rest—which may limit their preparatory potential.

From a mechanistic standpoint, performing exercise during the IPC reperfusion phase may potentiate IPC-induced adjustments through convergent vascular and metabolic pathways. The reperfusion period already increases shear stress and nitric oxide–mediated vasodilation, enhancing muscle perfusion (Tinken et al. 2010; Joyner and Casey 2015). Superimposing exercise at this time can further augment these hemodynamic responses while synchronizing mitochondrial and redox signaling activated by both reperfusion and exercise (Heusch 2015). This combination may improve the matching between oxygen delivery and utilization (Grassi et al. 2015) and promote a more effective physiological priming for subsequent high-intensity intermittent efforts. Nevertheless, if the exercise intensity or duration during reperfusion is excessive, it may counteract IPC benefits by inducing excessive oxidative stress or impairing vascular responsiveness (Heusch 2015). Since athletes commonly combine multiple strategies to enhance performance, we therefore reasoned that combining occlusion with exercise during reperfusion could produce synergistic effects by uniting the vascular and metabolic stimuli of ischemia–reperfusion with the neuromuscular and thermoregulatory priming induced by exercise.

Thus, the aim of this study was to evaluate whether active IPC—that is, intermittent exercise performed during the reperfusion phase (IPC-A)—can effectively enhance intermittent and high-intensity performance in young team sport players or influence physiological and perceptual variables. We hypothesized that IPC-A, in comparison with traditional (i.e., passive) IPC, would improve performance due to potential synergistic effects between IPC and exercise.

## Methods

### Participants and ethical care

Fifteen young male amateur basketball and soccer players (15 ± 1 years, 173 ± 7 cm, 66 ± 4 kg) served as participants in this study. All participants had been involved in regular training sessions (2–4 times per week, 1–2 h per session) for at least three years. During the study period, the participants were in the pre-season phase and not engaged in competitive matches. Their estimated V̇O₂_max_ was ≈ 44.5 ± 1.7 mL·kg^−1^·min^−1^ based on the distance covered (968 ± 201 m) in the YoYo Intermittent Recovery Test level 1 (YYIR1). Inclusion criteria were: (a) familiarized with YYIR1, and performed it at least three times in the last six months, (b) no smoking history during the last year, (c) absence of any cardiovascular or metabolic disease, (d) systemic blood pressure lower than 140/90 mmHg, and no use of antihypertensive medication, (e) no use of creatine supplementation, anabolic steroids, drugs or medication with potential effects on physical performance (self-reported) and (f) no recent musculoskeletal injury.

This study was approved by the local institutional Ethical Committee for Human Experiments (7.572.592). Participants and their parents both signed an informed consent form. Based on prior research (Bradley et al. 2011), a sample size smaller than 15 (i.e., *n* between six and nine) was sufficient to detect a significant (*p* < 0.05) difference among playing positions (group) in a similar test (YoYoIE2) (main dependent variable). Also, a study using a similar IPC protocol (independent variable) and physical test duration reported significant statistical effects with a sample size of 12 individuals (Cruz et al. 2015). To guide our sample size, an a priori power analysis was conducted using G*Power 3.1.9.6. For a repeated-measures ANOVA (within-subjects factors), assuming an effect size of f = 0.25 (medium), alpha = 0.05, power (1 − β) = 0.80, and four conditions, the required sample size was estimated at 12 participants. To mitigate the risk of potential dropouts and improve statistical robustness, we included 15 participants, resulting in a total of 60 experimental observations (i.e., four visits per participant).

### Research design

**Fig. 1:**
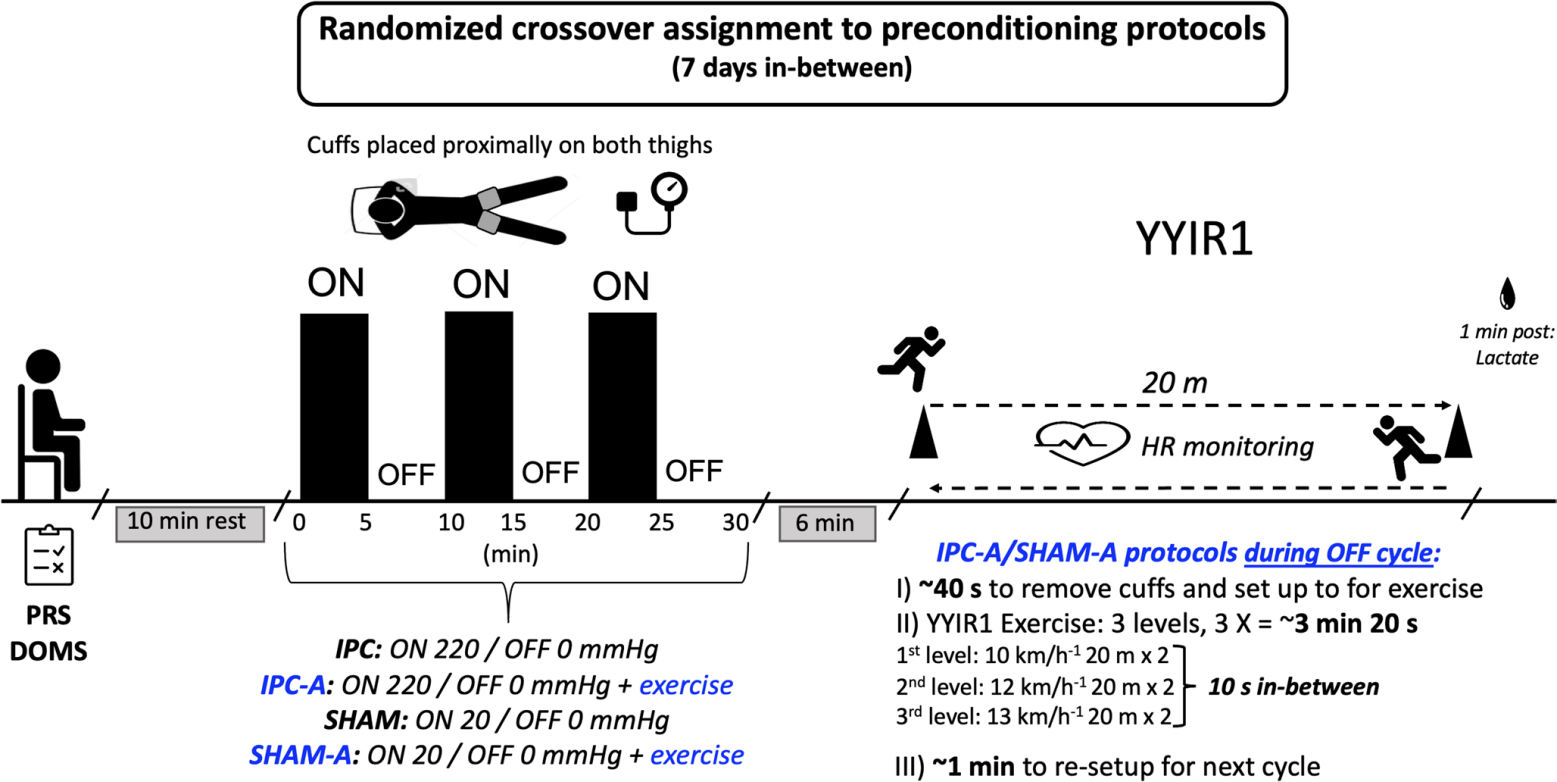
Overview of the experimental protocol. Fifteen participants completed the Yo-Yo Intermittent Recovery Test Level 1 (YYIR1) on four occasions, each following one of four protocols: traditional ischemic preconditioning at rest (IPC), active IPC (IPC-A), placebo at rest (SHAM), and active placebo (SHAM-A). Perceptual recovery status (PRS) and delayed onset muscle soreness (DOMS) were assessed at the start of each session. Heart rate (HR) was monitored during YYIR1; blood lactate were measured afterward. The YYIR1 tester was blinded to the prior protocol, and participants were blinded to performance data and study hypotheses

Figure 1 shows an overview of this study. Participants attended the laboratory on five occasions (7 days in-between). On the first visit, they underwent initial screening and anthropometric measurements, followed by familiarization with the equipment and proceedings. Perceptual recovery status (PRS) (Laurent et al. 2011) and perceptual delayed onset muscle soreness (DOMS) (Bijur et al. 2001) were assessed at the beginning of each session using a visual analogue scale while the participant was seated. From the second to fifth visits, they completed a preconditioning protocol, described below, following a randomized crossover assignment. Randomization was conducted using the online tool Randomizer (randomizer.org).

The YYIR1, described below, was carried out 6 min following the completion of each protocol. One minute after YYIR1, a blood sample was collected from the fingertip of the hand using an automatic retraction lancet, and lactate concentration was determined using a validated (Fell et al. 1998) portable analyzer (ROCHE Accu-Chek, Basel Switzerland). This time point was chosen based on evidence showing that blood lactate remains relatively stable during the first 6 min of recovery following the YYIR1 test (Krustrup et al. 2003). Heart rate (HR) was monitored throughout the entire YYIR1 by an individual HR monitor chest strap (RS800CX - Polar Electro^®^, Kempele, Finland). In the current study, the highest HR value in each condition has been referred to as Peak HR. All YYIR1 tests were conducted by the same experienced researcher at the same time of the day to avoid circadian variations. To prevent the possibility of a placebo (positive) effect, all participants were informed that all protocols could improve performance. To prevent nocebo (negative) effects, the participants were informed that IPC and SHAM would cause absolutely no harm, despite discomfort related to the maneuver (Marocolo et al. 2017). Additionally, the YYIR1 tester was blinded for which preconditioning protocol the participants had undergone immediately before. Also, the participants were kept blinded to their performance and other study data, such as HR and lactate levels, until the end of the research. To ensure blinding, we edited the audio so that stage numbers in the YYIRT1 were not heard. Participants refrained from coffee (or caffeine products), tea, alcohol intake as well as strenuous exercise for 24 h before testing.

### Ischemic preconditioning and placebo protocols

Four different protocols were compared: IPC (at rest), IPC-A, SHAM (at rest), and SHAM-A. The IPC maneuver consisted of three cycles of 5 min occlusion with a pressure of 220 mmHg and 5 min of reperfusion (no pressure) on both thighs, totalizing 30 min of intervention, using occlusion cuffs (12 cm wide x 86 cm length, rigid nylon material, ITS-MC™) positioned at the subinguinal region of the thighs. Due to a 15 s time window for manual cuff inflation to reach 220 mmHg of pressure, the procedure began at 4:45 min into the reperfusion phase of the IPC protocols.

The occlusion and reperfusion phases were conducted simultaneously in both thighs, with participants remaining in the lying position during occlusion. In the SHAM protocol, an external pressure of 20 mmHg was administered, as proposed in previous studies (Incognito et al. 2016; Marocolo et al. 2019).

### Active ischemic preconditioning and placebo protocols

The IPC-A and SHAM-A protocols were identical to IPC and SHAM, respectively, except that during the reperfusion phases, participants performed the first three levels of the YYIR1 test (10–13 km·h⁻¹; 20 m out and back with 10 s rest between levels). This short sequence (120 m) was repeated three times within each reperfusion phase, totaling ≈ 360 m per reperfusion phase. Each reperfusion phase was followed by 5 min of passive rest in the lying position (occlusion). The total accumulated distance across the three reperfusion phases was therefore 1080 m. This design ensured the exercise could be completed within the 5-minute reperfusion period, aligning with traditional IPC timing and allowing mild activation intended to elevate shear stress and metabolic flux without inducing fatigue. The selected exercise intensity and duration were sufficient to enhance nitric oxide–mediated vasodilation and muscle perfusion during reperfusion (Tinken et al. 2010; Joyner and Casey 2015), while minimizing metabolic disturbance (Heusch 2015). We acknowledge, however, that this standardized prescription may not have been optimal for all participants given interindividual differences in fitness level. Future studies could explore individualized exercise prescriptions based on physiological evaluations to optimize the active reperfusion stimulus.

IPC has been shown to improve exercise performance within 45 min of the final cuff inflation (Patterson et al. 2015). Accordingly, in the current study, YYIR1 was performed 6 min after the completion of the preconditioning protocol, ensuring that the test occurred within the optimal time window to preserve potential effects associated with the exercise performed during reperfusion.

### Yo-Yo intermittent recovery test level 1 (YYIR1)

The YYIR1 has been highly recommended for assessing endurance performance in intermittent sports, specifically focusing on the ability to perform intermittent exercise that maximally activates the aerobic system (Bangsbo et al. 2008). This test involves a 2 × 20-m shuttle run with increasing speed, starting at 10 km·h^− 1^. Participants were guided by audio cues, including a 10-second recovery period behind the finish line. The test was performed in an indoor sports court and ended when the participant failed to reach the lines on time twice (Bangsbo et al. 2008). The participants performed the YYIR1 individually to prevent influence from others, and the tester was unaware of the previous protocol performed. To standardize the conditions, the assessor provided no verbal encouragement or motivational cues during the test.

### Data analysis

We applied the Shapiro–Wilk test to verify the distribution of the data. For among-protocol analysis, a one-way analysis of variance (ANOVA) for repeated measures was conducted, followed by post-hoc Tukey’s test for parametric data. For nonparametric data, we applied the Friedman test followed by a post-hoc Dunn’s test. The significance level was set at 0.05. Also, to check individual data, we calculated the smallest worth change (SWC) approach contextualizing the IPC application to applied sports and exercise performance, as SWC = 0.6 x standard deviation (Marocolo et al. 2019).

## Results

There were no significant differences among the conditioning protocols in the scores of PRS (mean across the four sessions: 9.4 ± 1 a.u; *p* = 0.78) or DOMS (mean across the four sessions: 0.5 ± 1.3 a.u.; *p* = 0.78) at the beginning of the sessions.

**Fig. 2:**
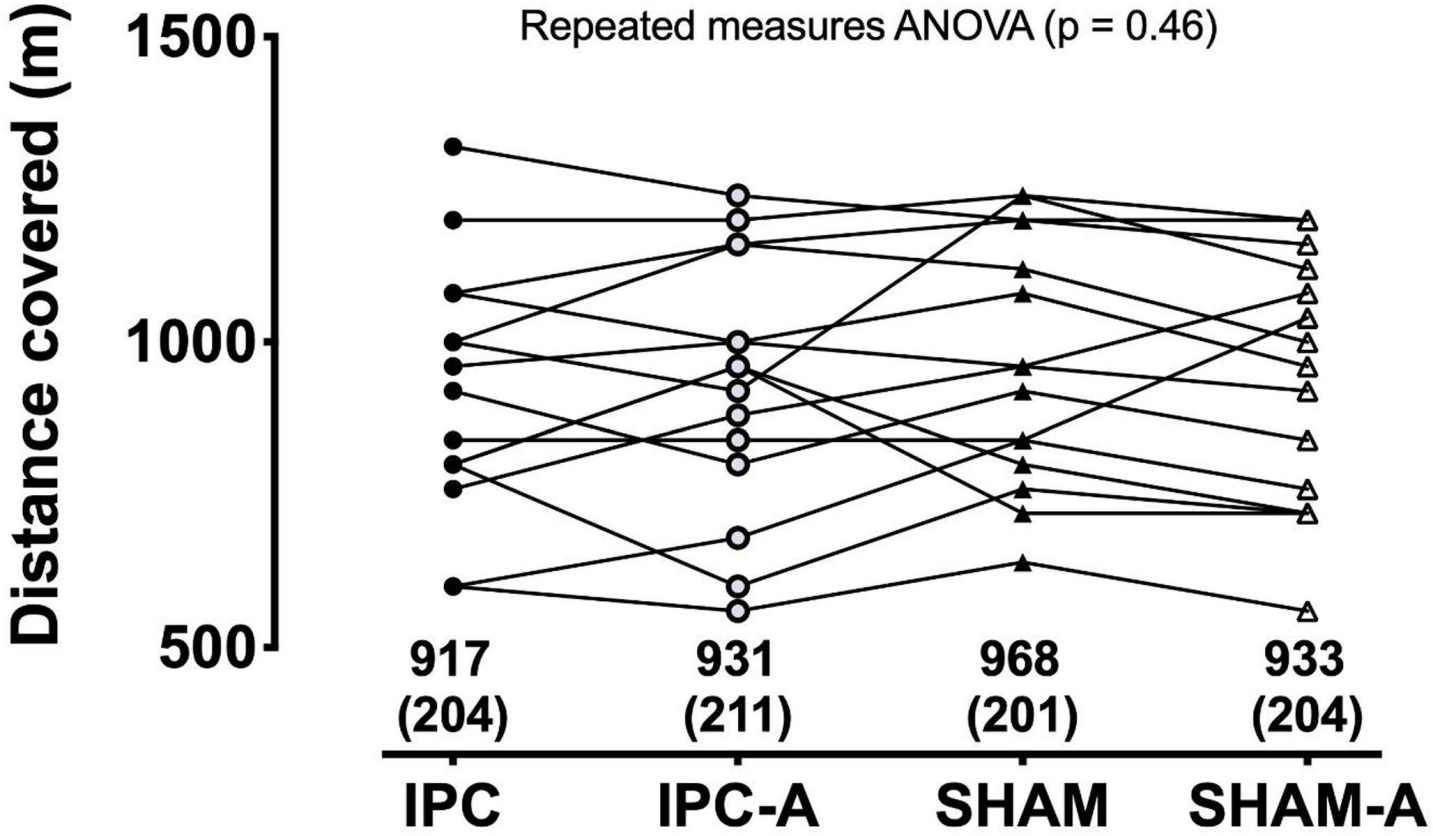
Distance covered during the YYIR1 among the different protocols. Values on the bottom are mean (standard deviation); Horizontal lines represent individual data; *n* = 15; IPC =  ischemic preconditioning; IPC-A = active ischemic preconditioning; SHAM = placebo; SHAM-A = active placebo

The YYIR1 distance covered was not different (*p* = 0.46) across all protocols (Fig. 2). The SWC value for YYIR1 distance covered was 120 m (0.6 × 201 - standard deviation of SHAM protocol). Table 1 provides the number of individuals that had better, worse, or unchanged performance after each protocol, compared to SHAM and considering the SWC.

**Table 1:** Number of participants that had a better, worse, or unchanged performance in the YYIR1 compared to SHAM, considering a SWC of ± 120 m

	BETTER	WORSE	UNCHANGED
IPC	1	5	9
IPC-A	2	4	9
SHAM-A	2	3	10

*n* = 15; IPC = ischemic preconditioning; IPC-A = active ischemic preconditioning; SHAM-A = active placebo

The HR responses during the exercise performed in the active protocols (i.e., IPC-A and SHAM-A) were ≈ 70% of the highest HR (peak HR) reached during the YYIRT in the respective protocols. Table 2 shows the HR responses during and blood lactate after the YYIR1. The peak HR values were lower (*P* = 0.01) during the YYIR1 preceded SHAM in comparison with SHAM-A. However, no differences (*P* = 0.22) were found for mean HR relative to the peak (%), as well as for blood lactate (*P* = 0.37) among the protocols (Table 2).

**Table 2:** Heart rate and blood lactate responses to the YYIR1

	IPC	IPC-A	SHAM	SHAM-A	*P*
Mean HR (bpm)	169 ± 8	172 ± 8	171 ± 7	174 ± 8	0.07
^#^ Peak HR (bpm)	193 ± 8	196 ± 9	194 ± 7	199 ± 9*	**0.01**
Mean HR (%HRpeak)	87 ± 1	88 ± 2	88 ± 2	87 ± 1	0.22
Blood lactate (mmol/L^− 1^)	9.5 ± 2.5	9.9 ± 3.0	9.5 ± 2.0	12 ± 4.0	0.37

HR = heart rate; bpm = beats per minute; values are mean ± standard deviation. *n* = 15; IPC = ischemic preconditioning; IPC-A = active ischemic preconditioning; SHAM = placebo; SHAM-A = active placebo; ^#^ the highest HR during the YYIR1 is peak HR; * higher than SHAM

## Discussion

This study evaluated the effects of an active IPC protocol (IPC-A) on high-intensity intermittent exercise performance in young team sport athletes, as well as its effects on physiological and perceptual variables. Although no performance benefits were observed, the use of an active protocol during reperfusion—a novel approach compared to traditional passive models—marks an important first step in refining IPC applications. Notably, the active distance prescribed during reperfusion may have exceeded the participants’ actual performance capacity, potentially diluting any ergogenic effect. Future studies might explore shorter or individualized active periods to enhance feasibility and effectiveness. Despite the neutral outcome, the rigorous blinding procedures employed strengthen the internal validity of our findings, contributing to the broader evidence base and supporting the publication of both positive and negative results (Marocolo et al. 2024).

In the present study, we adopted this specific IPC protocol because (a) ergogenic effects of IPC on exercise performance have been previously reported using this same approach (Jean-St-Michel et al. 2011; Cruz et al. 2015); (b) achieving a minimum ischemic stimulus duration of at least 4 min per cycle is essential, regardless of the number of ischemic repetitions (Ghosh et al. 2000) and, (c) it is considered safe and well tolerated in both patients and healthy individuals (Gonzalez et al. 2014). This rationale ensured that the applied protocol was both effective and practical for the current experimental design.

Incorporating a control is important to ensure consistent recovery status among participants executing the four distinct protocols: PRS and DOMS showed no significant differences among the protocols (*p* < 0.05), confirming that all protocols were carried out under identical recovery conditions.

The absence of significant differences in the distance covered during YYIR1 across all protocols (*p* = 0.46) and the mixed, inconclusive individual SWC results challenge our initial expectations. It should be acknowledged, however, that this interpretation assumes comparable acceleration and deceleration patterns across conditions, which may not perfectly reflect actual energy expenditure. Nonetheless, this assumption seems reasonable given the standardized nature of the YYIR1 (di Prampero et al. 2015; di Prampero and Osgnach 2018).

Despite the incorporation of exercise during the reperfusion phase in IPC-A, no clear performance enhancement emerged when compared to passive IPC. The specific exercise performed during the reperfusion phase (i.e., duration and intensity), as explored in our study, may have contributed to the observed lack of improvements. Future investigations should explore a spectrum of combinations involving different intensities and durations of exercise during reperfusion to better understand the potential benefits of IPC-A in high-intensity intermittent exercise among team sport players. In the context of studies evaluating IPC-A being limited, the array of potential combinations remains open, suggesting that the current findings, with mixed of individually meaningful SWC results, do not necessarily negate the possibility of IPC-A contributing to improvements in intermittent performance.

One study examined an IPC-A protocol in 12 men with a V̇O₂_max_ of ∼ 61 mL·kg^−1^·min^−1^. The authors conducted three trials, involving IPC-A (3 × 2 min cycling at ∼40 W with bilateral-leg cuff pressure of ∼180 mmHg), IPC at rest (4 × 5 min at 220 mmHg), and a SHAM procedure (4 × 5 min at < 10 mmHg). Subsequently, a 4 min maximal cycling performance test was performed. The study revealed superior performance in the 4 min maximal test following IPC-A compared to traditional (i.e., passive) IPC. The authors suggested that the performance-enhancing effect of IPC-A could be attributed to a placebo effect, improved pH regulation, and/or a change in the perception of effort (Christiansen et al. 2022). Of interest, the authors employed the IPC-A protocol with occlusion during exercise, while exercise occurred during the reperfusion phase in the present study.

In our study, which included basketball and soccer players, participants covered 968 ± 201 m, which is ≈ 37% less distance compared to soccer players of similar age (1538 ± 428 m) performing the same YYIR1 test (Markovic and Mikulic 2011). The inclusion of basketball players, whose game demands differ from soccer, likely contributed to the shorter YYIR1 distances observed in our mixed team sport sample. Given the participants’ relatively modest fitness level, the total distance covered during the active reperfusion phases of IPC-A (1080 m) may have represented a meaningful, yet not exhaustive, effort prior to the YYIR1 test. Since individuals with lower fitness levels tend to exhibit greater performance variability, detecting small effects—though still relevant—may be more challenging in these populations (Marocolo et al. 2018).

It is important to highlight that this was the first study to explore exercise during the reperfusion phase of an IPC protocol, and both active conditions (IPC-A and SHAM-A) involved the same exercise volume and structure. Therefore, any minor pre-fatigue would have affected both conditions equally and cannot explain the lack of difference between them. Future studies should test shorter or individualized active reperfusion bouts to further clarify the physiological contribution of this novel approach.

Overall HR and blood lactate values in the YYIR1, regardless of condition protocol, align with literature reports for adolescents and young team sport players (Krustrup et al. 2003; Souhail et al. 2010; Hammouda et al. 2013). However, regarding HR responses to IPC protocols, our findings contradict a previous study that reported decreased HR at the same power output during an incremental cycling test (Arriel et al. 2020), suggesting potential IPC benefits for endurance performance. In contrast, our results showed a higher peak HR during SHAM-A compared to SHAM (*p* = 0.01). The lower resting HR observed in SHAM (passive) relative to SHAM-A is expected, given the differing physiological demands; HR was likely already elevated at the start of the test in the SHAM-A condition. Importantly, despite these differences, no significant variations were observed in mean HR relative to peak HR (%) across protocols, highlighting the complex interaction between IPC, cardiovascular responses, and exercise performance. Similarly, blood lactate concentrations did not differ among protocols.

The present study adds novel evidence to the literature by systematically testing an active reperfusion model and underscores the value of reporting well-controlled neutral results to refine the understanding of IPC applications in applied exercise physiology. However, readers should consider the strengths and limitations of this study. A key strength lies in the novel design, which incorporated both active and passive IPC and SHAM protocols (IPC, IPC-A, SHAM, and SHAM-A), allowing for a comprehensive comparison of reperfusion strategies. Additionally, the study employed rigorous experimental controls to enhance internal validity (e.g., blinding assessors). Conversely, potential long-term effects (e.g., after multiple weeks of IPC-A) or the sustainability of performance improvements over extended periods were not assessed. The inclusion of additional measurements, such as oxygen consumption, muscle oxygenation, and rating of perceived exertion could have further elucidated the mechanisms underlying exercise responses to IPC. Although not a limitation per se, the young age of the participants may have influenced their responsiveness to IPC, as developmental factors can affect metabolic and performance responses (Armstrong et al. 2015). No formal assessment of biological maturation was conducted, which limits our ability to account for individual variability.

Additionally, it should be acknowledged that the active reperfusion protocol, although designed to enhance performance, may have imposed mild pre-fatigue in some participants, which could have influenced subsequent exercise responses. Furthermore, participants were moderately trained team sport athletes rather than elite, which may limit the generalizability of the findings to highly trained populations. Importantly, the paucity of IPC research in adolescent populations underscores the novelty and relevance of the present findings, contributing valuable insights to this underexplored area.

## Conclusion

We conclude that the application of an IPC-A protocol does not result in performance improvements or significant changes in blood lactate, and HR responses during high-intensity intermittent exercise in youth team sport players. It is essential to emphasize that the specificity of our conclusion is linked to unique aspects of the implemented IPC protocol, such as exercising during reperfusion phases and the precise duration/intensity of exercise, as well as the rigorous blinding procedures employed to enhance result reliability. Additionally, the relatively modest fitness level of the participants may have influenced the responsiveness to IPC-A, potentially limiting the detection of small effects that might be more apparent in highly trained athletes. Moving forward, future studies should explore a variety of combinations, incorporating different exercise durations, types, intensities, age groups, and fitness levels, to conduct a more comprehensive assessment of the potential benefits associated with an IPC-A approach.

## Data Availability

The data that support the findings of the current study are available from the corresponding author upon reasonable request.

## Abbreviations

ATP: Adenosine triphosphate
ANOVA: Analysis of variance
Bpm: Beats per minute
DOMS: Delayed onset muscle soreness
HR: Heart rate
IPC: Ischemic preconditioning
IPC-A: Active ischemic preconditioning
PRS: Perceptual recovery status
SHAM: Placebo protocol
SHAM-A: Active placebo protocol
SWC: Smallest worthwhile change
V̇O₂_max_: Maximal oxygen uptake
YYIR1: Yo-Yo Intermittent Recovery Test level 1
